# Evidence for energetic tradeoffs between physical activity and childhood growth across the nutritional transition

**DOI:** 10.1038/s41598-017-18738-4

**Published:** 2018-01-10

**Authors:** Samuel S. Urlacher, Karen L. Kramer

**Affiliations:** 1 Department of Anthropology, CUNY Hunter College, New York, NY 10065 USA; 2 Department of Anthropology, University of Utah, Salt Lake City, UT 84112 USA

## Abstract

Despite broad implications for understanding human life history, energetics, and health, the impact of physical activity on childhood growth remains unclear. Particularly understudied is the effect of secular changes in physical activity on child development. We address these shortcomings using data spanning the transition from traditional to semi-developed economy among Yucatec Maya agriculturalists. Anthropometric and behavioral observation data were collected from children living in a subsistence-based rural community in 1992 and again in 2012 following the introduction of a school and mechanized technologies but minimal overt dietary change. Multiple regression analyses demonstrate dramatic twenty-year transformations in how children spent their time. This behavioral change was associated with large declines in estimated physical activity level (PAL), associated activity energy expenditure savings of several hundred kilocalories/day, and sizable increases in mean height, weight, and triceps skinfold thickness. Controlling for observed frequency of market food consumption, PAL was inversely related to child body size and subcutaneous fat stores and significantly mediated the effects of data collection year on anthropometric indices. These findings indicate that physical activity can considerably influence childhood growth, highlighting the role of energy allocation tradeoffs between physical activity and competing life tasks in shaping patterns of human ontogeny and health.

## Introduction

Populations throughout the developing world are experiencing changes in economy and lifestyle^1,2^. This cumulative process of economic development, although context-specific, is reliably characterized by a suite of biological outcomes, including accelerated linear growth, younger age at reproductive maturation, greater incidence of overweight/obesity, and increased rates of metabolic and cardiovascular disease^3–9^. Identifying the precise aspects of economic development that underlie these trends in ontogeny, life history, and health has become a focus of human biology and epidemiology research^10–12^.

It is generally assumed that physical activity plays an important role in mediating the impact of economic development on human biology^13,14^. More specifically, it is understood that economic development is associated with a shift from an active ‘traditional’ to a sedentary ‘modern’ lifestyle that decreases individual physical activity energy expenditure (AEE). In combination with the displacement of customary diet by calorically dense and refined foods – increasing energy intake – this process of *nutritional transition* ^13^ is believed to result in positive energy balance and a surplus of calories that individuals either store as body fat or allocate directly to competing metabolic tasks (e.g., physical growth or reproduction).

While substantial advances have been made in understanding the dietary component of the nutritional transition^11,15,16^, remarkably little is known regarding the presumed role of physical activity in shaping the biological outcomes of economic development. This shortcoming is most salient for children, who contribute a large percentage of their overall energy budget to physical activity (averaging 29.0% at 10 years of age among Caucasians^17^), yet for whom reliable physical activity data spanning economic development are almost completely lacking^18,19^. Indeed, at the fundamental biological level, even basic relationships between physical activity and important aspects of child phenotype remain unclear. Although body size has important implications for human health and fitness^20,21^, it is largely unknown what impact physical activity has on child growth and nutritional status in non-industrialized contexts. This research limitation restricts understandings of energy use during childhood and the nature of variation in human ontogeny, life history, and health.

The present study addresses these shortcomings by investigating relationships between child free-living physical activity, body size and nutritional status, and early economic development among Yucatec Maya agriculturalists. We take advantage of a unique dataset consisting of anthropometric and behavioral observation data collected from children in a rural farming community in 1992 and again following substantial economic development – but minimal overt dietary change – in the same community in 2012. Four hypotheses are tested: (*Hypothesis* 1) Economic development is associated with a shift from high-energy-cost to low-energy-cost childhood physical activities, resulting in overall decreases in physical activity level (PAL) and AEE; (*Hypothesis* 2) Economic development is associated with accelerated physical growth and body fat accrual, leading to taller, heavier, and fatter children; (*Hypothesis* 3) Child PAL is inversely related to measures of body size and nutritional status, such that children spending a greater proportion of their overall energy budget on physical activity demonstrate more dramatic *tradeoffs* with indices of growth; and (*Hypothesis* 4) Child physical activity energetics mediate the relationship between economic development and growth, such that variation in PAL explains secular trends in child body size and nutritional status.

## Methods

### Study community

The Maya of the rural Yucatan community of Xculoc (*N* ≈ 500 individuals; hereafter referred to as the Yucatec Maya) have been the subject of economic, demographic, and life history research since the early 1990s^22,23^. In 1992, villagers were subsistence slash-and-burn maize agriculturalists with limited access to modern infrastructure. The community had no running water or electricity and no means for residents to regularly engage in formal education, health care, or wage labor. Child domestic and subsistence labor was critical to household economics and played an important role in underwriting high fertility^22,24,25^. Substantial community-wide changes have occurred since 1992, however, including the introduction of electricity, running water, a rudimentary health clinic, and a road connecting the community to the surrounding region. The establishment of a permanent school and the introduction of mechanized farming (both in the mid-2000s) have noticeably affected the everyday lives of children. Most children now regularly attend school, and completed education has increased from a mean of 4.5 years (*SD* = 2.8 years) in 1992 to 9.4 years (*SD* = 2.8 years) in 2012. The use of tractors and harvesters in agricultural production has reduced the demand for child labor.

Despite economic and lifestyle changes since 1992, informal ethnographic observation suggests that Yucatec Maya children continue to eat a predominately maize-based diet (accounting for ≈ 75–85% of total calories in 1992^22^) supplemented by traditional garden items, with minimal consumption of market foods or noticeable change in cooking style, portion sizes, or energy intake. This apparent dietary stability over the past twenty years provides an ideal context to isolate physical activity as an analytical predictor and observe *in situ* how secular changes in physical activity are related to child growth and nutritional status.

### Data collection

Data were collected from 155 Yucatec Maya children (age 4–10 years old) during the summer and fall of 1992 (*N* = 76 children) and 2012 (*N* = 79 children). Participants were recruited opportunistically and represent 89.4% and 92.9% of all eligible children in 1992 and 2012, respectively. Participant ages have been confirmed multiple times through annual censuses conducted since the 1990s. Parental informed consent with additional child informed assent was obtained from all participants. All methods and procedures were approved and conducted in accordance with guidelines set by community leaders and the Committee on the Use of Human Subjects Institutional Review Board of Harvard University (#18643).

Height, weight, and triceps skinfold thickness – indicating subcutaneous fat stores^26^ – were measured for all participants at study enrollment using conventional methods^27^. Body mass index (BMI, kg/m^2^) was calculated from height and weight data. Participants in 2012 provided additional subscapular and suprailiac skinfolds thickness measurements.

Behavioral observation data documenting physical activity and market food consumption (i.e., the consumption of commercially-produced food items) were collected from a subset of participants (*N*_1992_ = 25; *N*_2012_ = 45; recruited opportunistically) using well-validated instantaneous scan sampling techniques^22,28,29^. Household residential clusters were observed during 3- to 11-hour blocks spanning daylight hours from 7:00 am to 6:00 pm. During observation blocks, the specific activities of all participants were recorded simultaneously every 10 to 15 minutes using a hierarchical coding system that was developed for community-specific activities^22^. Participants were observed over a minimum of two complete 11-hour days.

### Data analysis

A total of 9,313 scan observations identified 186 unique physical activities and 70 instances of the consumption of market foods. Consumed market food items (*N* = 10) included rice, noodles, bread, milk, yogurt, gelatin, potato chips, candy (e.g., chocolate), sugary juice drinks, and soda. The use of cooking oil was not observed. For time allocation analysis, physical activities were grouped into six categories: *Work*, *Active Play*, *Education*, *Sedentary Leisure*, *Childcare*, and *Other* (Table 1). The amount of time (hours/day) a child spent in each category was calculated by multiplying the proportion of total observations spent in the given category by the 11-hour observation day. Activities performed simultaneously were attributed equal weight^22,30^. Market food consumption (items/day) was similarly calculated by averaging cases across 11-hour observation days. The quantity of foods consumed was not recorded, precluding estimates of energy intake.

**Table 1:** Time allocation activity categories and corresponding metabolic equivalent value (METs) characteristics.

Activity Category (*N* activities)	Description	METs^a^ Range	METs^a^ Mean (*SD*)
*Work* (*N* = 79)	Resource acquisition or domestic/agricultural labor. (Examples: retrieving water from well; sweeping floor; husking corn)	1.4–6.3	3.23 (0.93)
*Active Play* (*N* = 18)	Active, non-productive behavior. (Examples: chasing others; riding bicycle; playing soccer)	1.5–8.8	4.12 (1.51)
*Education* (*N* = 21)	Schooling, study, or direct transfer of knowledge. (Examples: listening to school lesson; doing homework; learning to sew)	1.2–6.6	2.32 (1.32)
*Sedentary Leisure* (*N* = 36)	Sedentary, non-productive behavior or personal maintenance. (Examples: watching television; resting; combing hair)	0.9–2.8	1.47 (0.43)
*Childcare* (*N* = 25)	Active or passive care of other children. (Examples: carrying baby; walking toddler; watching younger sibling)	1.3–2.8	2.98 (1.24)
*Other* (*N* = 7)	Activities not falling into any other category. (Examples: receiving medical care; accompanying adult)	1.2–3.8	1.93 (0.68)

^a^METs calculated from the activity-specific compendia of Ridley *et al*.^41^ and Ainsworth *et al*.^42^.

Indices of child physical activity energetics – AEE and PAL – were calculated from scan sampling data using the factorial method^31–33^. The factorial method assigns metabolic costs to specific activities using published reference values determined by calorimetry. It produces estimates of free-living energy expenditure within 3–8% of those obtained with gold standard doubly labeled water methods^34–36^ and has been widely used to assess individual- and population-level PAL and AEE among developing populations^30,32,37–40^. Although reliant upon reference information (typically from industrialized populations) and generally less accurate than more objective approaches, the factorial method permits estimation of physical activity energetics in 1992 – prior to the common use of doubly labeled water and wearable activity monitors. A detailed description of our factorial methodology, more rigorous than standard protocols, is provided in the supplementary information. Briefly, each of the 186 physical activities observed during the study was assigned a unique metabolic equivalent value (METs, a multiple of resting metabolic rate) using the child-specific compendium of Ridley *et al*.^41^ or, when needed, the adult compendium of Ainsworth *et al*.^42^. Participant PAL was then calculated by averaging METs across the 24-hour day. Child AEE (kcal/day) was calculated by multiplying PAL by estimates of resting energy expenditure (kcal/day) obtained using the age-, sex-, and weight-specific prediction equations of Schofield^43^.

Data were assessed for outliers, distributional normality, and other parametric modeling assumptions. To account for age- and sex-related differences in physical development, z-scores were calculated for height (HAZ), weight (WAZ), BMI (BAZ), triceps skinfolds (TSZ), subscapular skinfolds (SSZ), and suprailiac skinfolds (SIZ) using the Third National Health and Nutrition Examination Survey (NHANES III) growth references^44^, as has been recommended for the Maya^45^.

Multiple linear regression models were constructed to investigate differences between 1992 and 2012 in child time allocation, market food consumption, physical activity energetics, and indices of growth (*Hypotheses 1* and *Hypothesis 2*), the nature of relationships between these measures (*Hypothesis 3*), and the potential mediating role of physical activity in the relationship between year of data collection and body size (*Hypothesis 4*). Physical activity level, rather than AEE, was used as an index of physical activity energetics in analyses testing *Hypothesis 3–4* to account for size related differences in individual resting energy expenditure^32,46^. Preliminary models included age and, when appropriate, sex, year of data collection, market food consumption, and their interaction terms as covariate predictors. Log-likelihood ratio tests and Akaike’s information criteria were used to assess model fit and determine final model parameters. Interaction terms involving age and market food consumption were not significant in any preliminary analysis (all *p* > 0.1) and were removed from all final models. Statistical analyses for *Hypotheses 1–3* were performed in R 3.3.2 (http://cran.us.r-project.org/). To test *Hypothesis 4*, multiple regression causal mediation models were created using the *PROCESS* macro^47^ in SPSS 23.0 (IBM; Armonk, NY). Mediation models were bias-corrected using a 1,000 bootstrap resampling procedure. Statistical significance was established for all analyses at a level of *p* < 0.05.

### Data accessibility

The datasets and code supporting this article are available from the corresponding author on reasonable request.

## Results

### Secular changes in time allocation, physical activity energetics, and market food consumption

Dramatic differences in child time allocation were identified between 1992 and 2012 for both sexes and for all physical activity categories except *Other* (Table 2; complete models in Supplementary Table S1; Supplementary Fig. S1). Children in 2012 spent significantly less time in *Work*, *Active Play*, and *Childcare* and significantly more time in *Education* and *Sedentary Leisure* (all *p* < 0.01). Females had a larger twenty-year decline than males in time spent in *Childcare* (β = 0.63, *SE* = 0.27, *p* = 0.023), but a smaller decline in time spent in *Active Play* (β = −1.93, *SE* = 0.64, *p* = 0.004). No sex differences were observed for changes in time spent in *Work*, *Education*, *Sedentary Leisure*, or *Other* (all *p* > 0.1).

**Table 2:** Time allocation, physical activity energetics, and diet measures (mean, *SD*) in 1992 and 2012 by sex. Twenty-year change (20yrΔ) in each measure was modeled controlling for age. PAL = physical activity level; AEE = activity energy expenditure.

	Females			Males		
	1992 (N = 14)	2012 (N = 20)	20yrΔ	1992 (N = 11)	2012 (N = 25)	20yrΔ
Age (years)	7.14 (1.90)	7.55 (1.66)	—	7.15 (1.48)	8.02 (1.64)	—
*Time Allocation* ^a^						
Scan Observations (n)	144.5 (10.3)	128.0 (26.5)	—	138.4 (9.6)	128.3 (26.3)	—
Work (hrs/day)	1.94 (0.95)	0.50 (0.49)	−1.50***	0.96 (0.86)	0.25 (0.27)	−0.96***
Active Play (hrs/day)	4.21 (1.88)	3.16 (1.36)	−0.82*	7.22 (2.39)	3.55 (1.09)	−2.86***^2^
Education (hrs/day)	1.57 (0.67)	3.48 (2.14)	+1.75**	1.12 (1.06)	3.72 (1.44)	+1.93***
Sedentary Leisure (hrs/day)	1.71 (0.72)	3.47 (1.03)	+1.75***	1.02 (0.26)	3.28 (0.98)	+2.47***
Childcare (hrs/day)	1.06 (1.04)	0.07 (0.16)	−1.02***	0.31 (0.65)	0.03 (0.07)	−0.38**^1^
Other (hrs/day)	0.51 (0.26)	0.32 (0.35)	−0.16	0.37 (0.34)	0.18 (0.22)	−0.20
*Physical Activity Energetics*						
PAL	1.92 (0.12)	1.61 (0.12)	−0.31***	2.25 (0.18)	1.76 (0.15)	−0.49***^1^
AEE (kcal/day)^b^	769 (94)	536 (87)	−238***	1080 (153)	740 (164)	−402***^1^
*Diet*						
Market Foods (items/day)	0.24 (0.34)	0.52 (0.44)	+0.22	0.20 (0.22)	0.46 (0.43)	+0.30

^a^Data collected between 7:00 a.m. and 6:00 p.m.; ^b^Estimated from PAL using the resting energy expenditure prediction equations of Schofield^43^; *p < 0.05; **p < 0.01; ***p < 0.001; ^1^Significant sex difference in 20yrΔ at *p* < 0.05; ^2^Significant sex difference in 20yrΔ at *p* < 0.001.

Large and highly significant twenty-year decreases were also detected for PAL and AEE (Table 2; Supplementary Table S2). Mean PAL fell 0.49 units for males and 0.31 units for females between 1992 and 2012 (both *p* < 0.001). This reduction resulted in decreases of 402 and 238 kcal/day in mean estimated AEE for males and females, respectively (both *p* < 0.001). Male twenty-year declines were significantly larger than those of females for both PAL (β = −0.17, *SE* = 0.07, *p* = 0.021) and AEE (β = −135.8, *SE* = 63.6, *p* = 0.037).

In contrast to patterns observed for time allocation and physical activity energetics, no significant twenty-year changes were detected in market food consumption for males (*p* = 0.364), females (*p* = 0.493), or both sexes combined (β = 0.30, *SE* = 0.23, *p* = 0.183) (Table 2; Supplementary Table S1).

### Secular changes in body size and nutritional status

Significant twenty-year increases were identified for all child anthropometric measures except BMI and BAZ (Table 3). Controlling for age, males in 2012 were, on average, 6.46 cm (*SE* = 1.10, *p* < 0.001) or 1.23 z-scores (*SE* = 0.20, *p* < 0.001) taller, 2.60 kg (*SE* = 0.78, *p* < 0.001) or 0.54 z-scores (*SE* = 0.14, *p* < 0.001) heavier, and had triceps skinfolds that were 1.45 mm (*SE* = 0.64, *p* = 0.027) or 0.43 z-scores (*SE* = 0.14, *p* = 0.004) larger than their counterparts in 1992. Similarly, females in 2012 were 8.10 cm (*SE* = 1.06, *p* < 0.001) or 1.49 z-scores (*SE* = 0.20, *p* < 0.001) taller, 3.42 kg (*SE* = 0.65, *p* < 0.001) or 0.78 z-scores (*SE* = 0.14, *p* < 0.001) heavier, and had triceps skinfolds that were 1.42 mm (*SE* = 0.52, *p* = 0.008) or 0.37 z-scores (*SE* = 0.13, *p* = 0.006) larger than females twenty years earlier. No significant sex differences were observed for these twenty-year changes (all *p* > 0.1).

**Table 3:** Growth and nutritional status measures (mean, *SD*) in 1992 and 2012 by sex. Twenty-year change (20yrΔ) in each measure was modeled controlling for age. No statistically significant sex differences in 20yrΔ were detected.

	Females			Males		
	1992 (N = 38)	2012 (N = 41)	20yrΔ	1992 (N = 38)	2012 (N = 38)	20yrΔ
Age (years)	7.01 (1.91)	7.77 (1.77)	—	7.31 (1.92)	7.79 (1.64)	—
*Raw Measures*						
Height (cm)	102.27 (9.83)	114.52 (10.17)	+8.10***	106.10 (10.54)	114.86 (9.07)	+6.46***
Weight (kg)	16.27 (3.30)	21.36 (5.46)	+3.42***	18.24 (3.55)	21.75 (5.70)	+2.60**
BMI (kg/m^2^)	15.45 (1.39)	16.02 (1.54)	+0.45	16.07 (1.09)	16.23 (1.96)	+0.11
Triceps Skinfolds (mm)	7.72 (1.80)	9.48 (2.64)	+1.42**	7.18 (2.09)	8.57 (3.30)	+1.45*
Subscapular Skinfolds (mm)^a^	—	7.19 (2.62)	—	—	6.33 (2.67)	—
Suprailiac Skinfolds (mm)^a^	—	7.45 (3.18)	—	—	6.25 (3.39)	—
*NHANES III Z-Scores* ^b^						
Height-for-age	−3.20 (1.05)	−1.76 (0.69)	+1.49***	−3.04 (0.80)	−1.83 (0.94)	+1.23***
Weight-for-age	−1.35 (0.85)	−0.76 (0.57)	+0.78***	−1.59 (0.43)	−1.10 (0.75)	+0.54***
BMI-for-age	−0.10 (0.81)	0.01 (0.53)	+0.23	0.20 (0.62)	0.16 (0.70)	+0.03
Triceps-for-age	−0.56 (0.57)	−0.22 (0.54)	+0.37**	−0.48 (0.70)	−0.12 (0.62)	+0.43**
Subscapular-for-age^a^	—	0.02 (0.46)	—	—	0.11 (0.63)	—
Suprailiac-for-age^a^	—	−0.03 (0.65)	—	—	0.08 (0.71)	—

^a^Data available for 2012 only; ^b^Age- and sex-specific z-scores calculated using NHANES III growth references^44^; *p < 0.05; **p < 0.01; ***p < 0.001.

### Relationships between physical activity and growth

Relationships between measures of time allocation, PAL, and growth did not differ by sex or year of data collection in any model (all *p* > 0.1). Thus, estimates of effects were determined for the complete dataset. Models using only 1992 or 2012 data – demonstrating the consistency of relationships between PAL and growth indices within individual data collection years (i.e., independent of community-level economic development and possible changes in diet) – are provided in Supplementary Fig. S2.

**Figure 1:**
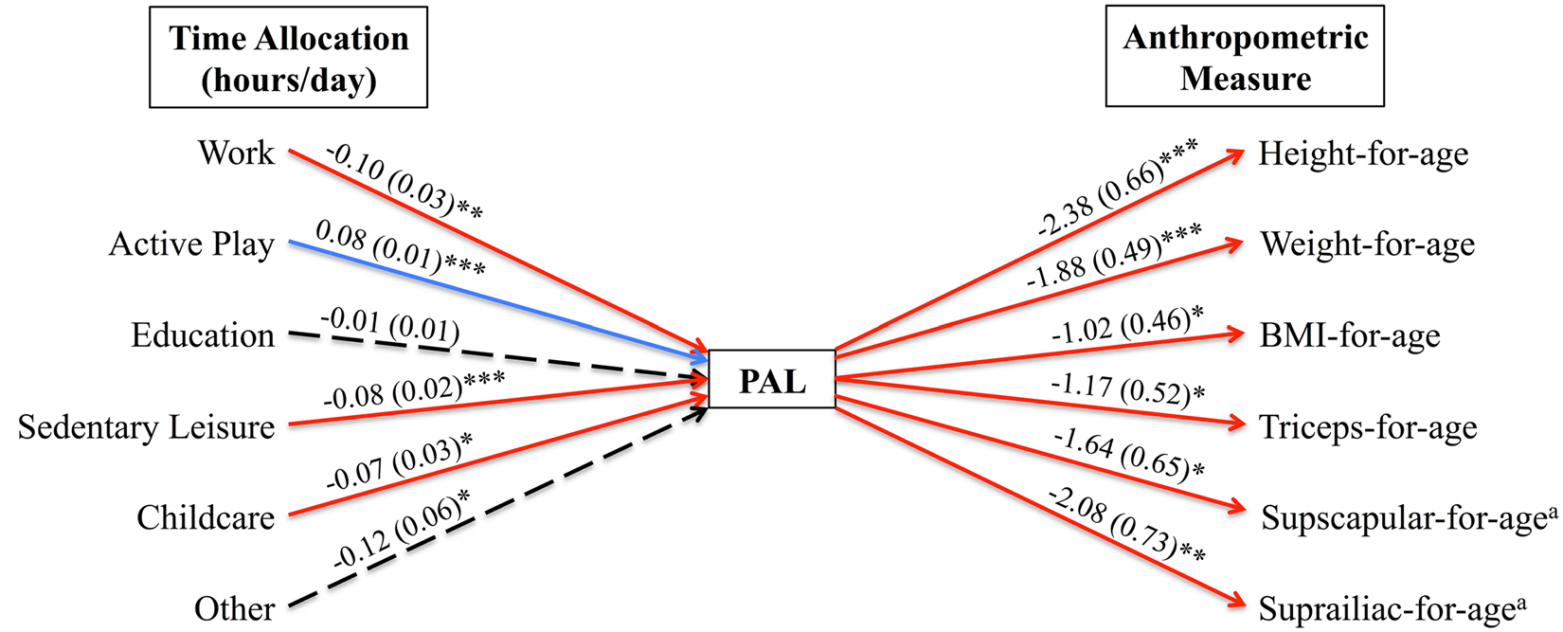
Results (*β*, *SE*) from multiple regression models testing relationships between time allocation factors and physical activity level (PAL, left) and PAL and anthropometric indices of growth and nutritional status (right). Relationships did not significantly differ by sex or year in any model (all *p* > 0.1). Solid red line = significant negative relationship; Solid blue line = significant positive relationship; ^a^Data available for 2012 only; *p < 0.05; **p < 0.01; ***p < 0.001.

Time spent in specific activity categories was closely related to estimates of PAL (Fig. 1), such that PAL was positively predicted by time spent in *Active Play* (*p* < 0.001) but negatively predicted by time spent in *Work* (*p* < 0.001), *Sedentary Leisure* (*p* < 0.001), and *Childcare* (*p* = 0.026). Time spent in *Education* (*p* = 0.466) and *Other* (*p* = 0.057) had no significant effect on PAL.

**Figure 2:**
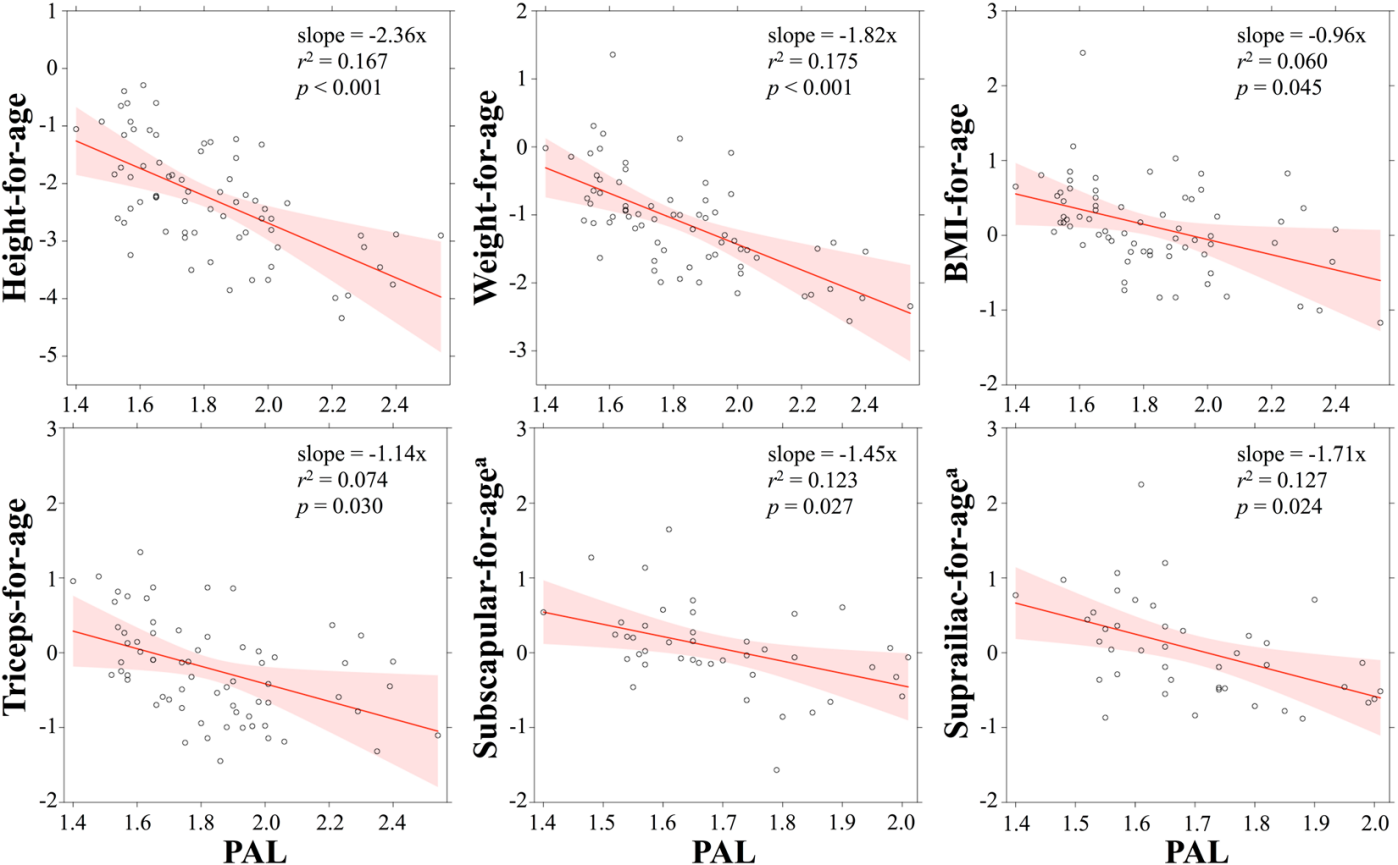
Results from multiple regression models (estimates, 95% confidence intervals) illustrating relationships between physical activity level (PAL) and indices of growth and nutritional status. ^a^Data available for 2012 only.

In turn, models controlling for age, sex, year of data collection, and market food consumption demonstrate that PAL was negatively related to HAZ (*p* < 0.001), WAZ (*p* < 0.001), BAZ (*p* = 0.032), and TSZ (*p* = 0.027), such that children spending a greater percentage of their overall energy budget on physical activity were shorter, lighter, and had lower levels of subcutaneous fat stores than their less active peers (Fig. 1; Supplementary Table S3). Among children in 2012, PAL was also inversely related to the additional body fat measures of SSZ (*p* = 0.016) and SIZ (*p* = 0.007). The size of these PAL effects on indices of growth and nutritional status were substantial. A one standard deviation increase in PAL (0.25 units) from the overall sample mean, for example, was associated with a decrease of 0.60 units in HAZ, 0.47 units in WAZ, 0.26 units in BAZ, and 0.29 units in TSZ. For the typical 7.5-year-old participant, this equates to body size decreases of 3.17 cm, 2.01 kg, 0.48 kg/m^2^, and 1.0 mm, respectively. Partial residual plots illustrating the amount of total variance explained by PAL in each model (range = 7.0 to 18.3%) are provided in Fig. 2. Market food consumption, entered as a covariate, did not significantly predict anthropometry in any model (all *p* > 0.07).

### Physical activity mediation of the impact of year of data collection on growth

**Figure 3:**
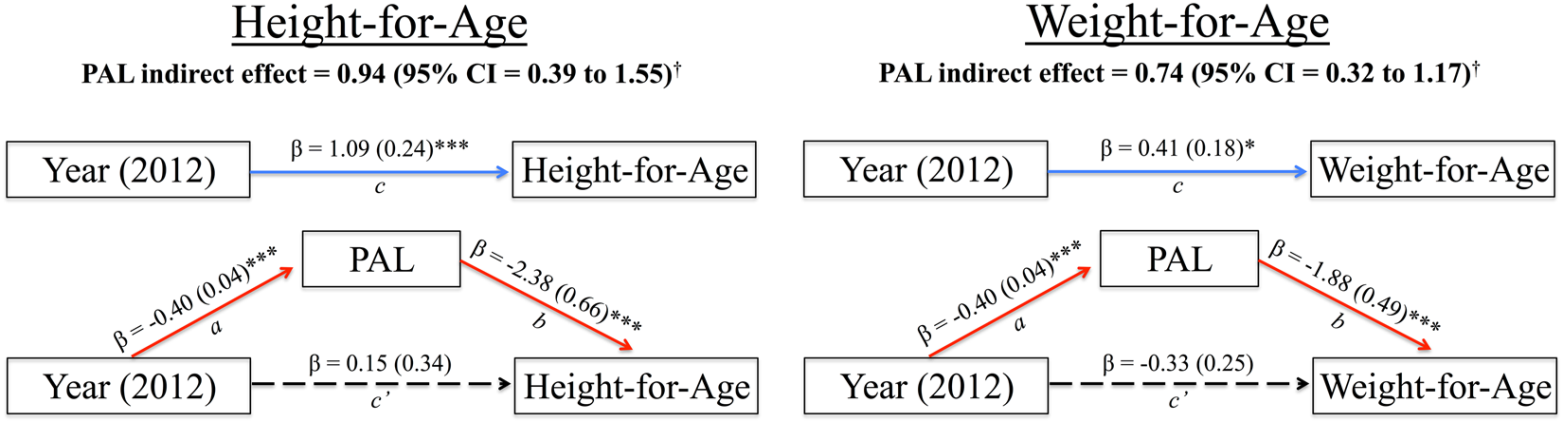
Causal mediation models illustrating the statistically significant mediating role of physical activity level (PAL) in relationships between year of data collection (1992 vs. 2012) and child height-for-age (left) and weight-for-age (right). Solid red line = significant negative relationship; Solid blue line = significant positive relationship ^a^Independent effect of year on PAL; ^b^Independent effect of PAL on height/weight; ^c^Total effect of year on height/weight; ^c’^Independent (direct) effect of year on height/weight; *p < 0.01; ***p < 0.001; ^†^Significant mediating role of PAL indicated by bias-corrected 95% confidence intervals that do not contain zero.

Causal mediation models controlling for age, sex, and market food consumption indicate that PAL plays a statistically significant underlying role in the relationship between year of data collection and child anthropometric measures (Fig. 3). Significant indirect effects of PAL in the relationships between year of data collection and HAZ (coefficient = 0.94, 95% CI = 0.39–1.55) and WAZ (coefficient = 0. 74, 95% CI = 0.32–1.17), in fact, exhibit complete statistical mediation^47^, such that the significant total positive effects of year of data collection on HAZ (*p* < 0.001) and WAZ (*p* = 0.023) are reduced to non-significant direct effects (*p* = 0.653 and *p* = 0.201, respectively) when including PAL as a predictor in each model. Thus, significant overall relationships between year of data collection and HAZ and WAZ are driven by the mediating effects of PAL. Total effects of year of data collection on BAZ (β = −0.03, *SE* = 0.15, *p* = 0.829) and TSZ (β = 0.19, *SE* = 0.17, *p* = 0.266) did not reach significance in the subset of participants for whom PAL data are available and, as such, mediation models for these measures are not reported.

## Discussion

This study provides a unique repeated cross-sectional analysis of childhood physical activity, body size, and nutritional status spanning early stages of economic development. It also represents a rare investigation of the fundamental biological relationship between physical activity and subadult growth. A number of findings offer insight into the role of physical activity in shaping variation in human energy use, ontogeny, and health.

Child physical activity decreased dramatically over twenty years of economic development (*Hypothesis 1*). Following a suite of community-level changes, Yucatec Maya children in 2012 dedicated significantly more time each day than their counterparts in 1992 to activities relating to *Education* and *Sedentary Leisure* and significantly less time to *Work*, *Childcare*, and *Active Play*. This shift from high-energy-cost to low-energy-cost behaviors resulted in significant reductions in estimated PAL and associated AEE savings of several hundred kilocalories per day. These findings provide strong *in situ* evidence for a negative relationship between economic development and child physical activity and physical activity energetics. As such, results corroborate the implication of secular trends in child physical activity drawn from limited cross-cultural and urban-rural comparisons^18,37,48,49^. During early stages of economic development, these trends appear closely related to the introduction of technology and infrastructure that simultaneously reduce the value of and demand for child economic participation (a complete list of observed *Work* activities is provided in Supplementary Table S4) while providing sedentary alternatives to traditional forms of active play. The important role of consumer electronics in this phenomenon is evident; Yucatec Maya children in 2012 spent on average 1.05 hours/day (*SD* = 0.75 hours/day) watching television, a sedentary activity not available to community members in 1992 and recognized to lower child PAL throughout the developing world^18,50^.

In addition to illuminating secular trends in childhood physical activity, this study provides evidence for dramatic increases in child growth and more modest increases in nutritional status accompanying economic development (*Hypothesis 2*). Yucatec Maya children in 2012 were on average 7.3 cm (7%) taller, 3.0 kg (17%) heavier and had triceps skinfold measures of subcutaneous body fat that were 1.4 mm (19%) greater than those of children in 1992. These findings are consistent with large increases in child body size and fatness following early economic development elsewhere among the Maya^51,52^ and other populations globally^6,53–55^. We note that only one child in the present sample was overweight by international standards^56^. However, anthropometric data reported for nearby urban-living Maya^57^ and those living in the United States^58^ suggest that advancing economic development may soon lead to the emergence of prevalent overweight/obesity and metabolic dysfunction. Identifying the underlying socio-ecological factors driving this trend is critical to developing effective health policy to battle the ongoing pandemic of childhood chronic disease^59^.

Synthesizing behavioral and anthropometric datasets, results of the present study suggest that habitual physical activity is an important factor driving variation in childhood growth and nutritional status (*Hypothesis 3*). Yucatec Maya children who spend a greater percentage of their overall energy budget on physical activity are significantly shorter, lighter, and have lower levels of subcutaneous fat stores than their less active peers, irrespective of observed frequency of market food consumption. The strength of these relationships is illustrated by large effect sizes, the observation that PAL explains up to 18% of total variation in indices of growth and nutritional status, and the broad consistency of negative relationships between PAL and anthropometric measures within individual data collection years. Critically, PAL significantly mediates the effects of year of data collection on child height and weight (*Hypothesis 4*), indicating that secular change in growth among the Yucatec Maya may be largely explained by concurrent reductions in physical activity energetics. We note that estimated mean decreases in AEE between 1992 and 2012 (females = −238 kcal/day, males = −402 kcal/day) can alone easily offset the predicted energetic cost of growing (4,780 kcal/kg^60^, distributed across multiple years) and maintaining (41 kcal/kg/day^45^) increases in Yucatec Maya body size over the same period (females = +3.42 kg, males = +2.60 kg). Together, these findings support the widely held but largely untested assumption that childhood physical activity is involved in the nutritional transition^13,14^ and complement numerous studies in the developing world demonstrating the impact of dietary change on subadult body size (e.g.,^6,7^). In agreement with informal ethnographic observation of diet, market food consumption was rare in the present sample of children, and no difference was found in the frequency of observed market food consumption between 1992 and 2012. This finding, together with non-significant relationships between market food consumption and anthropometry in all examined models, implies that diet does not currently play a large role driving variation in Yucatec Maya growth. However, this analysis is limited by inability to quantify energy intake, and differences in diet almost certainly explain growth variability to some degree. Future research among children must utilize direct measures of energy expenditure and energy intake, as well as longitudinal measures of growth, to further investigate these causal relationships.

Life history theory provides a useful framework for understanding the negative relationship between childhood physical activity and growth. Children, particularly those living in non-industrialized settings, have energy budgets that are constrained by factors such as small digestive tracts, brain development, and high levels of immune activity^61–63^. In such contexts, life history theory predicts that energy allocation *tradeoffs* occur between life’s competing metabolic tasks in a manner that maximizes fitness^64–66^. Any decrease in habitual physical activity should, thus, free calories for alternative biological use. The findings of the present study suggest that a substantial portion of energy liberated from physical activity during childhood is reallocated toward the generation of soma in the form of body growth and, to a lesser degree, fat deposition. This interpretation is consistent with research in animal models, including mice^67^ and free-living Assamese macaques^68^. It also conforms to a number of studies in the developing world demonstrating that child manual labor is associated with reduced height, weight, and BMI^69–71^. As predicted by life history theory, the relationship between habitual physical activity and growth is less apparent in contexts of relative energy abundance^72,73^. However, child physical activity maintains an inverse relationship with body fat among industrialized populations^12,74,75^. The physiological pathways mediating these tradeoffs – likely via the growth hormone/insulin-like growth factor-1 axis^76,77 ^– represent an important target for future investigation.

One particularly interesting question that emerges from the present study is why Yucatec Maya children, despite what has been suggested for nutritionally stressed hunter-gatherers^40,78^, do not reduce physical activity to low overall levels in order to minimize AEE and promote greater growth. One potential explanation surrounds the adaptive value of play. Although work-related behavior is typically the focus of studies investigating child physical activity energetics among non-industrialized populations (e.g.^79,80^), active play is by far the most expensive and pervasive element of child AEE profiles in the present sample. This finding underscores the ontogenetic and evolutionary importance of play behavior^81^. For humans, play is believed to promote, among other things, the skill acquisition necessary to successfully navigate complex social systems and master difficult methods of resource acquisition^82–84^. Thus, the fitness advantages of expending energy in play may at times outweigh the possible advantages of greater body growth^68^. One alternative or complementary hypothesis is that larger body size itself, while often associated with greater fitness among humans^20,21^, is not advantageous in all contexts. Although children facing severe malnutrition may reduce physical activity to preserve energy for anabolic processes^85^ or future reproduction^40^, this study suggests that some children with modest nutritional status maintain high levels of physical activity at a direct cost to growth. The possible fitness advantages of smaller, rather than larger, body size are varied and include lower recurring physiological maintenance costs and reduced resource competition with siblings^64,86^. Among the Yucatec Maya, high natural fertility rates^87^ suggest that short stature during adulthood does not incur a group-level fitness handicap. Detailed analysis of the potential costs and benefits of body size during childhood, however, is needed.

## Conclusions

This study contributes to a mounting body of research suggesting that physical activity is an important determinant of human ontogeny, life history, and health^9,88,89^. Among Yucatec Maya agriculturalists, physical activity appears to exhibit strong energetic tradeoffs with childhood growth and body fat deposition, with such tradeoffs spanning early economic development and underlying the ongoing nutritional transition. These findings are consistent with evolutionary life history theory and provide insight into the fundamental biological mechanisms driving human phenotypic variation. Future research must validate and build on this work by incorporating direct energetic and dietary measures into childhood behavioral analysis among developing populations.

## Electronic supplementary material

Supplementary Information
