# Supplementary material for: Evidence for energetic tradeoffs between physical activity and childhood growth across the nutritional transition
Source: Sci Rep. 2018 Jan 10;8:369. doi: 10.1038/s41598-017-18738-4 (PMC5762677; doi:10.1038/s41598-017-18738-4)
Supplement: Supplementary file 1 — Supplementary Information [file 41598_2017_18738_MOESM1_ESM.pdf]

Supplementary Information:

Evidence for energetic tradeoffs between physical activity and childhood growth across the nutritional transition

Samuel S. Urlacher*[1] and Karen L. Kramer[2]

[1]Department of Anthropology, CUNY Hunter College, New York, NY 10065
[2]Department of Anthropology, University of Utah, Salt Lake City, UT 84112

Factorial Method Protocol Detail

In total, 186 unique child physical activities were observed over the course of the study. Each of these activities was assigned a published metabolic equivalent value (MET) representing an activity-specific caloric cost as a multiple of individual resting metabolic rate. Child-specific METs from the compendium of Ridley et al. (2008) were available for 52 activities. All other activities were assigned METs from the adult compendium of Ainsworth et al. (2011), following recommended methods for the handling of such data (Harrell et al., 2005; Ridley and Olds, 2008). This protocol of assigning energy expenditure values to specific physical activities (e.g., ‘washing dishes’) is more rigorous than the common practice of assigning blanket METs across a small number of general activity categories (e.g. ‘domestic work’). Given our scan sampling approach, METs were assigned for the full duration of any single 10-15 minute scan observation (i.e., children observed husking corn during a scan performed once every 10 minutes were assigned the MET specific to husking corn for a full 10 minutes, regardless of possible differences in actual [unobserved] duration of husking corn over this timeframe). This limitation in quantifying the time spent in specific activities is a source of error in our analysis. Direct observation data were not collected on school grounds in 1992. As such, the energetic cost of activity during rare school attendance in 1992 was estimated as the average METs associated with school activity in 2012 (2.4 METs). To calculate conventional 24-hour energetic measures, evening and early morning (i.e., 6:00 pm to 7:00 am) activity falling outside of observation time was also estimated. Each participant was assigned 10 hours of sleep per day (Torun, 2005) at a value of 0.9 METs (Ridley et al., 2008). The remaining three hours of unobserved time per day (involving predominantly indoor activity as Maya children rarely go outside after dark) were assigned a value of 1.2 METs, equivalent to the mean METs for indoor activity at all times in the complete dataset. Final estimates of individual PAL were obtained by calculating average METs across 24-hour days.

References

Ainsworth BE, Haskell WL, Herrmann SD, Meckes N, Bassett J, David R, Tudor-Locke C, Greer JL, Vezina J, Whitt-Glover MC, Leon AS. 2011. 2011 Compendium of Physical Activities. Medicine & Science in Sports & Exercise 43(8):1575-1581.

Harrell JS, McMurray RG, Baggett CD, Pennell ML, Pearce PF, Bangdiwala SI. 2005. Energy Costs of Physical Activities in Children and Adolescents. Medicine & Science in Sports & Exercise 37(2):329-336.

Ridley K, Ainsworth BE, Olds TS. 2008. Development of a Compendium of Energy Expenditures for Youth. International Journal of Behavioral Nutrition and Physical Activity 5(1):45.

Ridley K, Olds TS. 2008. Assigning Energy Costs to Activities in Children. Medicine & Science in Sports & Exercise 40(8):1439-1446.

Torun B. 2005. Energy requirements of children and adolescents. Public Health Nutrition 8(7a):1-26.

Table S1. Parameter estimates from final multiple linear regression models investigating twenty-year changes in child time allocation and diet by sex.

	Females		Males	
Model	*β (SE)*	*p*-value	*β (SE)*	*p*-value
Work (hrs/day)				
Age (years)	0.18 (0.06)	0.009	0.16 (0.05)	0.002
Year (2012)	-1.50 (.23)	< 0.001	-0.96 (0.18)	< 0.001
Active Play (hrs/day)				
Age (years)	-0.63 (0.11)	< 0.001	-0.52 (0.14)	0.001
Year (2012)	-0.82 (0.40)	0.047	-2.86 (0.54)	< 0.001
Education (hrs/day)				
Age (years)	0.42 (0.15)	0.009	0.43 (0.12)	0.001
Year (2012)	1.75 (0.54)	0.003	1.93 (0.46)	< 0.001
Sedentary Leisure (hrs/day)				
Age (years)	0.18 (0.09)	0.842	-0.14 (0.09)	0.122
Year (2012)	1.75 (0.33)	< 0.001	2.47 (0.32)	< 0.001
Childcare (hrs/day)				
Age (years)	0.08 (0.07)	0.232	0.07 (0.04)	0.067
Year (2012)	-1.02 (0.24)	< 0.001	-0.38 (0.14)	0.009
Other (hrs/day)				
Age (years)	-0.07 (0.03)	0.020	0.00 (0.03)	0.876
Year (2012)	-0.16 (0.10)	0.118	-0.20 (0.11)	0.070
Market Foods (items/day)				
Age (years)	-0.17 (0.09)	0.075	-.01 (0.39)	0.897
Year (2012)	0.22 (0.32)	0.493	0.30 (0.33)	0.364

Table S2. Parameter estimates from final multiple linear regression models investigating twenty-year changes in child physical activity energetics by sex.

	Females		Males	
Model	*β (SE)*	*p*-value	*β (SE)*	*p*-value
Physical Activity Level				
Age (years)	-0.01 (0.01)	0.265	-0.00 (0.02)	0.920
Year (2012)	-0.31 (.04)	< 0.001	-0.49 (0.06)	< 0.001
Activity Energy Expenditure (kcal/day)				
Age (years)	11.5 (8.8)	0.198	40.7 (15.6)	0.014
Year (2012)	-237.7 (31.2)	< 0.001	-402.3 (58.9)	< 0.001

Table S3. Parameter estimates from final multiple linear regression models investigating relationships between physical activity level (PAL) and anthropometric indices of growth and nutritional status.

Model	*β (SE)*	*p*-value
Height-for-age		
PAL	-2.38 (0.66)	< 0.001
Age (years)	-0.12 (0.06)	0.033
Sex (male)	0.44 (0.237)	0.066
Year (2012)	0.14 (0.33)	0.678
Market Foods (items/day)	0.04 (0.11)	0.701
Weight-for-age		
PAL	-1.88 (0.49)	< 0.001
Age (years)	-0.18 (0.04)	< 0.001
Sex (male)	0.14 (0.17)	0.433
Year (2012)	-0.31 (0.24)	0.213
Market Foods (items/day)	0.12 (0.08)	0.139
BMI-for-age		
PAL	-1.02 (0.46)	0.032
Age (years)	-0.10 (0.04)	0.017
Sex (male)	0.50 (0.17)	0.004
Year (2012)	-0.47 (0.23)	0.049
Market Foods (items/day)	0.13 (0.08)	0.100
Triceps-for-age		
PAL	-1.17 (0.52)	0.027
Age (years)	-0.05 (0.05)	0.253
Sex (male)	0.55 (0.19)	0.005
Year (2012)	-0.30 (0.27)	0.264
Market Foods (items/day)	0.06 (0.09)	0.478
Subscapular-for-age		
PAL	-1.64 (0.65)	0.016
Age (years)	0.02 (0.05)	0.674
Sex (male)	0.46 (0.20)	0.027
Market Foods (items/day)	0.12 (0.11)	0.279
Suprailiac-for-age		
PAL	-2.08 (0.73)	0.007
Age (years)	-0.06 (0.06)	0.294
Sex (male)	0.62 (0.23)	0.010
Market Foods (items/day)	0.23 (0.12)	0.070

Table S4. Complete list of Work activities recorded during behavioral observation ($N = 79$).

**Work Activity**	**Work Activity (continued)**
Applying pesticide in garden	Picking up chopped wood
Bends corn stalks in garden	Placing item on fire
Burning waste	Planting in garden
Chopping brush in garden	Pounding maize
Chopping wood	Processing seeds for planting in garden
Collecting small items	Refilling sack with fertilizer in garden
Collecting wood	Repairing (carpentry)
Cooking	Repairing (shoe)
Delivering food (upon request)	Retrieving on bicycle (light)
Delivering non-food item (upon request)	Retrieving water from pila
Digging hole in yard (upon request)	Retrieving water from toma
Discarding waste	Retrieving water from well
Dumping water	Returning wood to home (walking)
Embroidering by hand	Running errand in town (not carrying)
Exchanging food with neighbor (upon request)	Running errand to neighboring town (not carrying)
Extinguishing fire	Selling item
Feeding domesticated animal	Serving food
Fetching corn from mill	Sewing by hand
Fetching item from neighbor (upon request)	Sharpening pencil
Fetching load from garden	Shelling corn
Filling bucket with water	Shooing domesticated animal
Folding clothes	Sorting clothes
Gardening work (general)	Spraying herbicide in garden
Going to borrow item	Swatting domesticated animal
Going to procure item	Sweeping floor
Going to purchase item (upon request)	Sweeping road
Grinding corn	Taking down hammock (upon request)
Harvesting in garden	Transferring items (upon request)
Hunting	Unloading/putting away item
Husking corn	Using machete to clear garden
Killing domesticated animal	Walking to garden
Loading wood	Walking from garden
Looking for item (following instruction)	Walking in garden
Making digging stick (upon request)	Walking to collect wood
Mending by hand	Washing clothes
Mixing sascab	Washing corn
Moving line in garden	Washing dishes
Organizing/putting items away (walking)	Washing floor
Organizing/putting items away (standing)	Weeding by hand
Peeling corn	

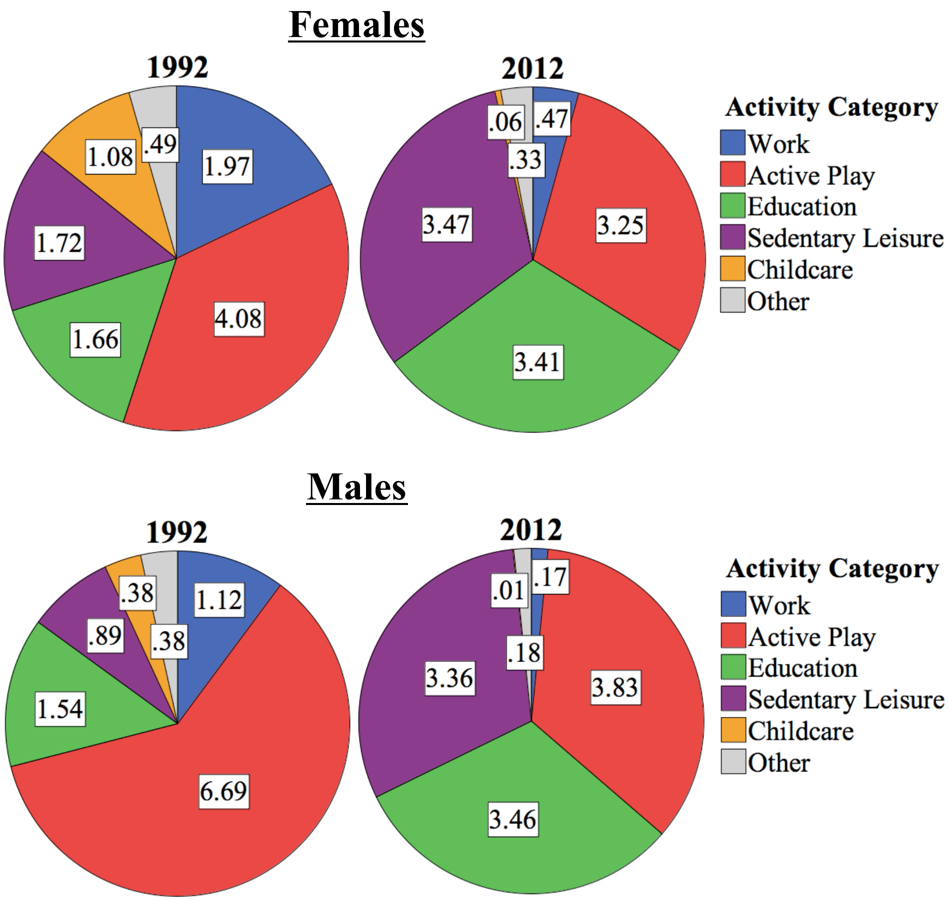

Figure S1. Time allocation profiles (marginal mean hours/day) for females (top) and males (bottom) in 1992 (left) and 2012 (right) estimated from linear regression models controlling for age. Significance tests for twenty-year change in each activity are provided in Table 2.

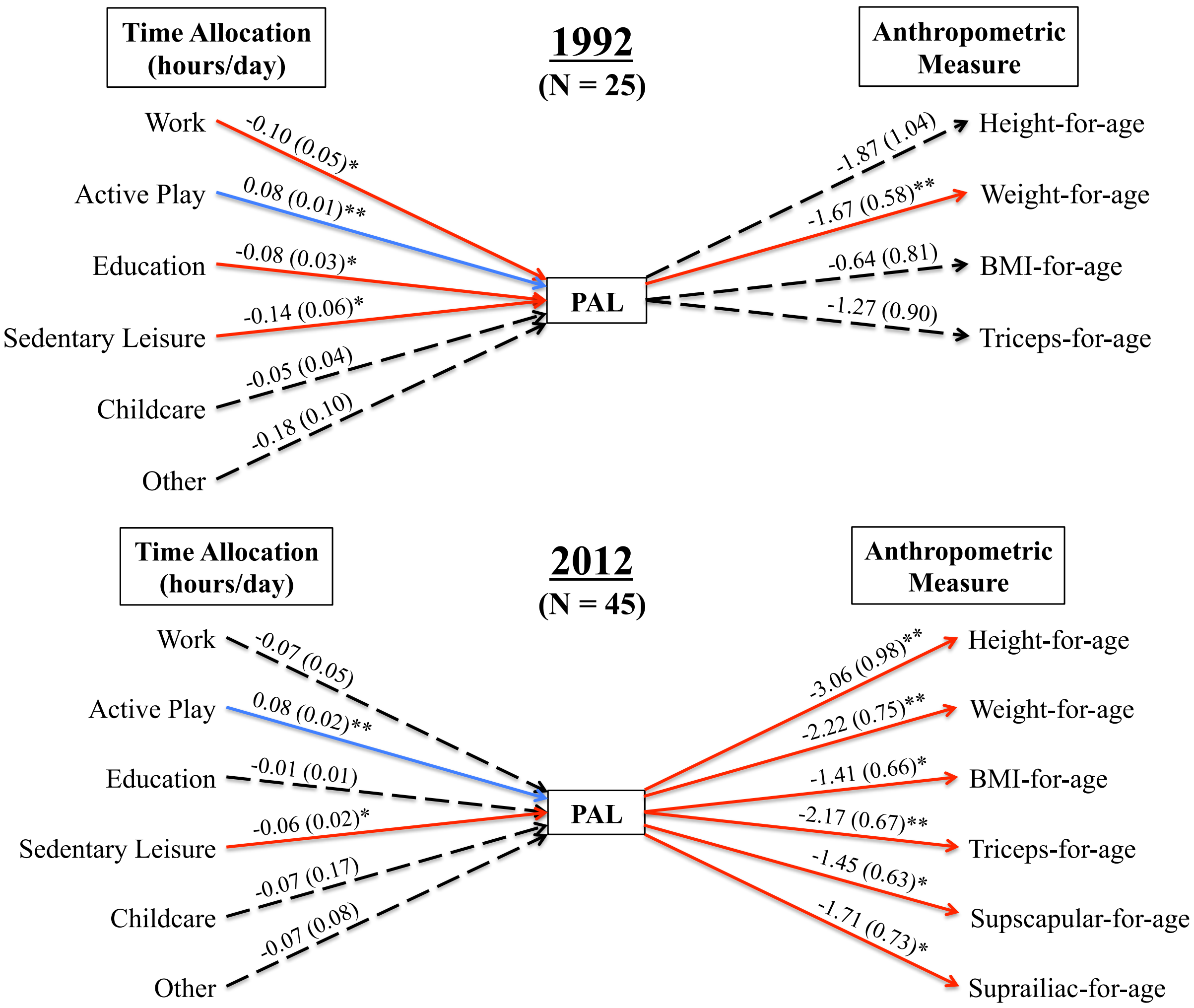

Figure S2. Results (β, SE) from multiple linear regression models testing relationships between child time allocation factors and physical activity level (PAL, left) and PAL and anthropometric indices of growth and nutritional status (right) in 1992 (top) and 2012 (bottom). Models control for age, sex, and market food consumption. Relationships did not significantly differ by sex in any model (all $p > 0.1$). Solid red line = significant negative relationship; solid blue line = significant positive relationship; * $p < 0.05$; ** $p < 0.01$.
